# Development and validation of a predictive model for cervical insufficiency incorporating AMH and androstenedione

**DOI:** 10.1038/s41598-025-31678-8

**Published:** 2025-12-13

**Authors:** Xueqing Zhao, Mingyue Ma, Yuanying Liu, Yue Ma, Rong Li, Yongqing Wang

**Affiliations:** 1 Department of Obstetrics and Gynecology, Peking University Third Hospital, Beijing, 100191 China; 2 National Clinical Research Center for Obstetrics and Gynecology, Peking University Third Hospital, Beijing, 100191 China; 3 State Key Laboratory of Female Fertility Promotion, Department of Obstetrics and Gynecology, Peking University Third Hospital, Beijing, 100191 China; 4 Key Laboratory of Assisted Reproduction, Peking University, Ministry of Education, Beijing, 100191 China; 5 Beijing Key Laboratory of Reproductive Endocrinology and Assisted Reproductive Technology, Beijing, 100191 China; 6 Department of Community Health Sciences, UIC School of Public Health, Chicago, USA

**Keywords:** Cervical insufficiency, IVF–ET, Risk factor, Prediction, Predictive model, Androstenedione, Anti-müllerian hormone, Risk factors, Endocrine reproductive disorders

## Abstract

This study aims to develop a predictive model for cervical insufficiency (CI) in women who undergo in vitro fertilization and embryo transfer (IVF-ET) based on relevant indicators measured prior to pregnancy. A total of 2,494 women who received IVF-ET at the Reproductive Medical Center of the Third Hospital of Peking University between 2016 and 2022 were included. All participants ultimately delivered at the same institution. 1,745 patients were assigned to the training cohort and 749 to the validation cohort. Both univariate logistic regression analysis and multiple logistic regression analysis were conducted to establish the CI prediction model. Among the 2,494 cases, the incidence rate of CI was 3.2%. Risk factors identified to be associated with CI included body mass index (BMI) > 22.83 kg/m^2^, testosterone (T) level > 0.74 nmol/L, androstenedione (A) level > 11.45 nmol/L, anti-Müllerian hormone (AMH) level > 3.50 ng/ml, frequency of hysteroscopic surgery, number of previous pregnancies (gravidity), and pre-pregnancy diabetes. Cervical length > 3.15 cm is a protective factor for cervical insufficiency. Conversely, factors such as the endometrial preparation regimen, occurrence of an intrauterine operation within six months before pregnancy, and the uterine length were not found to be significant risk factors for CI. The area under the curve (AUC) for this model achieved 0.819, with a 95% confidence interval of 0.758 to 0.881. Despite the lack of external validation from independent cohorts, this study successfully developed a comprehensive predictive model for CI in women undergoing IVF-ET, providing a preliminary exploration for early prediction and intervention strategies.

**Supplementary Information:**

The online version contains supplementary material available at 10.1038/s41598-025-31678-8.

## Introduction

Cervical insufficiency (CI) is characterized by painless dilation or shortening of the cervix during the second trimester of pregnancy^1^, with incidence ranges between 1% and 3%^2,3^。CI constitutes 8–9% of preterm births, 8 ~ 25% of extremely preterm births, and 8 ~ 25% of recurrent miscarriages in the second and third trimesters^4^.

Diagnosis typically relies on a patient’s medical history, physical examinations, ultrasound evaluations during pregnancy.In some cases, preconception cervical function tests are also required^3,5,6^. Identifying CI among women without a history of cervical insufficiency or who have not undergone cervical function tests can be very challenging since many diagnoses were made when ultrasound results suggest the occurrence of cervical shortening or dilatation, which usually happens during the second trimester and leads to poorer pregnancy outcomes^7^. For instance, some women may experience premature rupture of membranes or intrauterine infections due to cervical dilation, potentially missing opportunities for timely clinical intervention and resulting in miscarriage or preterm delivery. In some cases, surgical intervention, such as emergency cerclage surgery, may be required. In one study, the abortion rate after emergency cervical cerclage was reported to be 20.89%, with premature birth rates reaching 68.35%^8^. These figures are notably higher than those associated with preventive cerclage surgery^9^.This implies that at present, women with CI often experience at least one adverse pregnancy outcome before receiving a diagnosis, delaying preventive treatments aimed at improving future pregnancy outcomes.

Even so, there are currently few established prediction methods or early diagnostic approaches for CI in clinical practice. There are only a limited number of retrospective studies in which risk factors associated with CI, most of the results have been limited to PCOS and testosterone levels^10,11^, with the occasional study discussing the correlation between past medical history and CI, unfortunately without combining PCOS and testosterone levels^12^. Thus, these studies considered only a restricted set of factors in their analyses, leading to a constrained predictive effect. Research into the aetiology of CI remains relatively rare and incomplete. Consequently, developing a more comprehensive and accurate method for the early prediction of CI is imperative.

The aim of the current study is to explore the risk factors associated with CI in individuals undergoing IVF-ET. To achieve this, a predictive model for CI using information obtained from women prior to pregnancy will be constructed. Given that these women undergo more comprehensive physical examinations before conception, we can obtain more detailed and extensive data to construct a more comprehensive and accurate predictive model.

## Materials and methods

### Study population

The study population comprised women who achieved successful pregnancies through IVF-ET at the Reproductive Center of Peking University Third Hospital between January 2016 and November 2022. These women subsequently delivered at the hospital’s Obstetrics Department. Comprehensive data were collected for all cases, covering preconception and pregnancy periods. Women with a history of cervical treatments-including cervical resection, cervical conization, loop electrosurgical excision procedures, laser treatment, and cryotherapy-or with female genital malformations were excluded. Ultimately, 2,494 cases were included in the analysis. Of these, 1,745 cases constituted the training set for model development, while 749 cases were allocated to the validation set for assessing the model’s performance.

### Data collection

The data collected for this study included various variables, such as age; prepregnancy body mass index (BMI); prepregnancy hormone levels, including follicle-stimulating hormone (FSH), luteinizing hormone (LH), oestradiol(E2), testosterone (T), androstenedione (A), and anti-Müllerian hormone (AMH); diagnosis of polycystic ovary syndrome (PCOS); embryo cryopreservation (covering both fresh and frozen embryo transfers); the controlled ovarian stimulation protocol used in the fresh embryo transfer cycle (including clomiphene (CC), ultralong GnRH agonist, long GnRH agonist, short GnRH agonist, and standard GnRH agonist protocols); endometrial preparation method in the frozen embryo transfer cycle (natural or artificial cycles); frequency of hysteroscopic surgeries; frequency of curettage procedures; time elapsed between the last intrauterine procedure and pregnancy; previous gravidity (G) and parity (P); twin pregnancy status; prepregnancy hypertension and diabetes; and measurements of uterine and cervical length before the use of ovulation-inducing medications. All the data were extracted from the electronic medical records system of Peking University Third Hospital.

Further specifications were applied to certain variables. Hormone levels were assessed during the initial visit and on days 2–5 of the menstrual cycle; for participants with irregular menstrual cycles, such as those with PCOS, hormone testing was conducted on any day of the cycle. Additionally, to maintain consistency, women with hereditary collagen synthesis disorders, including Marfan syndrome or Ehlers–Danlos syndrome^13,14^, were excluded, as these conditions are recognized as risk factors for CI. Finally, in this study, multiple pregnancies were classified strictly as twin pregnancies, with cases of triplet or higher-order pregnancies excluded. The cervical length is measured by ultrasound physician as the distance from the internal os to the external os of the cervix and is reported in the ultrasound report.

### Diagnostic criteria for CI

The currently recommended diagnostic criteria for CI are as follows^3,5,6^: (1) history-based diagnosis: defined as two or more instances of painless cervical dilatation resulting in recurrent miscarriage in the second trimester or preterm birth with painless cervical dilatation, after excluding other causes; (2) ultrasound-based diagnosis: which requires a history of one or more pregnancy losses or preterm births and a cervical length ≤ 25 mm before 24 weeks of gestation; and (3) physical examination-based diagnosis: identified by painless cervical dilatation or prolapsed fetal membranes detected on manual or speculum examination before 24 weeks of gestation.

### Sample size considerations

The target sample size and the number of events per predictor were calculated using the R package “pmsampsize”, following the methodology outlined by Riley et al.^15^. The number of candidate predictors was specified as 8, with an event rate of 0.023 and a C-statistic of 0.9. The minimum required sample size for the training set was determined to be 1058, while the minimum number of events per predictor was estimated at 3.04, corresponding to a total of at least 25 events. Subsequently, considering a 70/30 training/validation split, the total sample size for the internal cohort was calculated, requiring a minimum of 1511 patients and 35 events.

### Data analysis

Data for this study were obtained from the electronic medical records system of Peking University Third Hospital. Collection and organization were conducted using Microsoft Excel 2020, and subsequent processing and analysis were performed in R (version 4.2.2). In the data, some variables have a certain degree of missing values: LH 39/2494 (1.56%), FSH 143/2494 (5.73%), E2 36/2494 (1.44%), P 80/2494 (3.21%), T 27/2494 (1.08%), A 35/2494 (1.40%), AMH 44/2494 (1.76%), uterine length 51/2494 (2.04%), and cervical length 52/2494 (2.09%). The missing values were filled using the multiple imputation method.

Descriptive statistics, including mean ± standard deviation (x ± s) were used to describe normally distributed measurement data, with intergroup comparisons conducted via the t-test. For non-normally distributed data, median and quartile values M (P25, P75) were used, and intergroup comparisons utilized the rank-sum test. Categorical variables are presented as sample numbers (%), with intergroup comparisons performed using the chi-square test or Fisher’s exact test.

To ensure unbiased analysis, participants were randomly divided into a training set (70%) and a validation set (30%) at a 7:3 ratio using the R sample function. The training set was utilized for model development, while the validation set was reserved for model performance verification. Optimal biomarker thresholds (e.g., BMI, LH/FSH ratio, E2, P, T, A, AMH levels, and uterine and cervical lengths) were determined using the Youden index (J = sensitivity + specificity − 1), which maximizes the combined sensitivity and specificity of the receiver operating characteristic (ROC) curve in training set. This approach prioritizes balanced classification performance for clinical risk stratification.

The model development was initiated with the training set. Univariate logistic regression analysis was first conducted to identify potential predictors of CI occurrence, with a significance level of *P* < 0.05. LASSO regression was then applied to screen for meaningful predictors. Multivariate logistic regression analysis was used to evaluate variable independence and construct the final prediction model. A nomogram was generated to visualize model outcomes. The model underwent 1,000 rounds of self-sampling validation to assess its robustness, with calibration evaluated via a calibration curve. Model discrimination was assessed through the area under the ROC curve (AUC). A clinical decision curve analysis (DCA) and Calibration curvewas conducted to quantify the net benefit across a range of threshold probabilities, evaluating the model’s clinical utility. Finally, the constructed multifactor model was validated using the validation set.

The “pROC” and “ggplot2” packages were used to plot the ROC curves. The “rms” package was used for nomogram construction and calibration plotting, and “ggDCA” functions were utilized for the DCA. All the statistical tests were two-tailed, with statistical significance defined as *P* < 0.05.

## Results

### Incidence rate of CI in women undergoing IVF-ET

A total of 2,494 women undergoing IVF-ET were included in this study. Among them, 80 cases of CI were identified, yielding an incidence rate of 3.2%. The prevalence of CI in the training set was 3.3%(57/1745), and that in the validation set was 3.1%(23/749). Detailed characteristics of the study population are presented in Table 1 and Supplement Table 1.

**Table 1:** Characteristics in CI and non-CI women.

	CI	non-CI	*P*
	*N* = 80	*N* = 2414	
Age, y	34.00 (32.00, 36.00)	34.00 (31.00, 36.00)	0.239
BMI, kg/m^2^, n (%)			< 0.001
≤22.83	35 (43.8)	1574 (65.2)	
>22.83	45 (56.2)	840 (34.8)	
LH/FSH, n (%)			0.004
​≤1.03	15 (18.8)	222 (9.2)	
​>1.03	65 (81.2)	2192 (90.8)	
E2, pmol/L, n (%)			0.341
​≤175.50	43 (53.8)	1426 (59.1)	
​>175.50	37 (46.2)	988 (40.9)	
P, nmol/L, n (%)			0.693
​≤1.19	41 (51.2)	1183 (49.0)	
​>1.19	39 (48.8)	1231 (51.0)	
T, nmol/L, n (%)			< 0.001
​≤0.74	33 (41.2)	1843 (76.3)	
​>0.74	47 (58.8)	571 (23.7)	
A, nmol/L, n (%)			< 0.001
​≤11.45	55 (68.8)	2259 (93.6)	
​>11.45	25 (31.2)	155 (6.4)	
AMH, ng/ml, n (%)			< 0.001
​≤3.50	35 (43.8)	1733 (71.8)	
​>3.50	45 (56.2)	681 (28.2)	
PCOS, n (%)			< 0.001
NO	50 (62.5)	2182 (90.4)	
YES	30 (37.5)	232 (9.6)	
Embryo cryopreservation, n (%)			0.487
Frozen embryo	40 (50)	1112 (46.1)	
Fresh embryo	40 (50)	1302 (53.9)	
Protocol of controlled ovarian stimulation/Endometrial preparation plan, n (%)			0.022
CC	0 (0)	3 (0.1)	
Ultralong GnRH agonist	8 (10.0)	179 (7.4)	
Long GnRH agonist	8 (10.0)	287 (11.9)	
Short GnRH agonist	0 (0)	20 (0.8)	
GnRH agonist	24 (30)	813 (33.7)	
Natural cycle	10 (12.5)	588 (24.4)	
Artificial cycle	30 (37.5)	524 (21.7)	
Frequency of hysteroscopic surgery	1.00 (0.00, 1.00)	0.00 (0.00, 1.00)	0.002
Frequency of curettage operation	0.00 (0.00, 1.00)	0.00 (0.00, 1.00)	0.026
Last intrauterine operation within 6 months from the last menstruation			0.003
No	42 (52.5)	941 (39.0)	
Yes	18 (22.5)	404 (16.7)	
No operation	20 (25.0)	1069 (44.3)	
G	1.00 (0.00, 2.00)	0.00 (0.00, 1.00)	< 0.001
P			0.367
0, n (%)	73 (91.2)	2263 (93.7)	
≧1, n (%)	7 (8.8)	151 (6.3)	
Twin pregnancy, n (%)			0.005
No	53 (66.2)	1916 (79.4)	
Yes	27 (33.8)	498 (20.6)	
Prepregnancy diabetes, n (%)			0.001
No	73 (91.2)	2369 (98.1)	
Yes	7 (8.8)	45 (1.9)	
Prepregnancy hypertension, n (%)			0.005
No	73 (91.2)	2355 (97.6)	
Yes	7 (8.8)	59 (2.4)	
Uterine length, cm, n (%)			0.004
​≤4.95	63 (78.8)	1525 (63.2)	
​>4.95	17 (21.2)	889 (36.8)	
Cervical length, cm, n (%)			0.149
​≤3.15	53 (66.2)	1404 (58.2)	
​>3.15	27 (33.8)	1010 (41.8)	

### Logistic regression analysis of High-risk factors for CI

The cut-off point determined by Youden index for BMI is 22.83 kg/m², for testosterone is 0.74 nmol/L, for androstenedione is 11.45 nmol/L, for AMH is 3.50 ng/mL, for cervical length is 3.15 cm.

In the training cohort of 1,745 cases, univariate regression analysis identified 14 high-risk factors associated with CI (Table 2). LASSO regression analysis of the above 14 factors narrowed the selection to 10 significant predictors (Supplement Table 2). Multivariate logistic regression analysis further revealed that the following factors were positively associated with the incidence of CI: BMI > 22.83 kg/m² (OR = 2.499, 95%CI: 1.388 ~ 4.572, *p* = 0.002), testosterone > 0.74 nmol/L (OR = 3.324, 95%CI: 1.846 ~ 6.048, *p* < 0.001), androstenedione > 11.45 nmol/L (OR = 4.410, 95%CI: 2.155 ~ 8.826, *p* < 0.001), AMH > 3.50 ng/ml (OR = 2.073, 95%CI: 1.093 ~ 3.928, *p* = 0.025), frequency of hysteroscopic surgery (OR = 1.576, 95%CI: 1.200 ~ 2.033, *p* = 0.001), previous gravidity (OR = 1.404, 95%CI: 1.072 ~ 1.818, *p* = 0.012), and prepregnancy diabetes (OR = 4.989, 95%CI: 1.472 ~ 14.429, *p* = 0.005). Conversely, a cervical length > 3.15 cm (OR = 0.365, 95%CI: 0.167 ~ 0.727, *p* = 0.007) was negatively associated with CI incidence (Table 3).

**Table 2:** Univariate regression analysis of the training cohort.

	OR(95%CI)	*P*
Age, y	1.026 (0.949, 1.107)	0.515
BMI > 22.83, kg/m^2^	3.063 (1.794, 5.343)	< 0.001
LH/FSH > 1.03	0.401 (0.214, 0.807)	0.006
E2 > 175.5, pmol/L	1.588 (0.935, 2.708)	0.087
*P* > 1.19, nmol/L	1.361 (0.799, 2.357)	0.262
T > 0.74, nmol/L	4.776 (2.797, 8.299)	< 0.001
A > 11.45, nmol/L	8.584 (4.731, 15.193)	< 0.001
AMH > 3.50, ng/ml	3.574 (2.099, 6.172)	< 0.001
PCOS	5.571 (3.129, 9.693)	< 0.001
Embryo cryopreservation		
Frozen embryo	reference	
Fresh embryo	0.678 (0.395, 1.150)	0.151
Protocol of controlled ovarian stimulation/Endometrial preparation plan		
Ultralong GnRH agonist	838466.216 (0.000, NA)	0.994
Long GnRH agonist	395236.600 (0.000, NA)	0.994
Short GnRH agonist	1.000 (0.000, 6519284347347.680)	1.000
GnRH agonist	379098.912 (0.000, NA)	0.994
Natural cycle	296006.985 (0.000, NA)	0.994
Artificial cycle	1040534.251 (0.000, NA)	0.993
Frequency of hysteroscopic surgery	1.555 (1.207, 1.971)	< 0.001
Frequency of curettage operation	1.375 (0.990, 1.835)	0.042
Last intrauterine operation within 6 months		
No	reference	
Yes	1.092 (0.553, 2.061)	0.792
No operation	0.417 (0.212, 0.781)	0.008
G	1.414 (1.109, 1.770)	0.004
P		
0	reference	
≧1	1.480 (0.507, 3.449)	0.414
Twin pregnancy	1.796 (0.981, 3.155)	0.048
Prepregnancy diabetes	5.556 (2.031, 12.929)	< 0.001
Prepregnancy hypertension	4.611 (1.699, 10.592)	0.001
Uterine length > 4.95, cm	0.695 (0.390, 1.201)	0.203
Cervical length > 3.15, cm	0.369 (0.175, 0.705)	0.005

**Table 3:** Multivariate regression analysis of the training cohort.

	OR(95%CI)	*p*
BMI > 22.83, kg/m^2^	2.499 (1.388,4.572)	0.002
Testosterone > 0.74, nmol/L	3.324 (1.846,6.048)	< 0.001
Androstenedione > 11.45, nmol/L	4.410 (2.155,8.826)	< 0.001
AMH > 3.50, ng/ml	2.073 (1.093,3.928)	0.025
Frequency of hysteroscopic surgery	1.576 (1.200,2.033)	0.001
Previous gravidity	1.404 (1.072,1.818)	0.012
Prepregnancy diabetes	4.989 (1.472,14.429)	0.005
Cervical length > 3.15, cm	0.365 (0.167,0.727)	0.007

### Prediction model

**Fig. 1:**
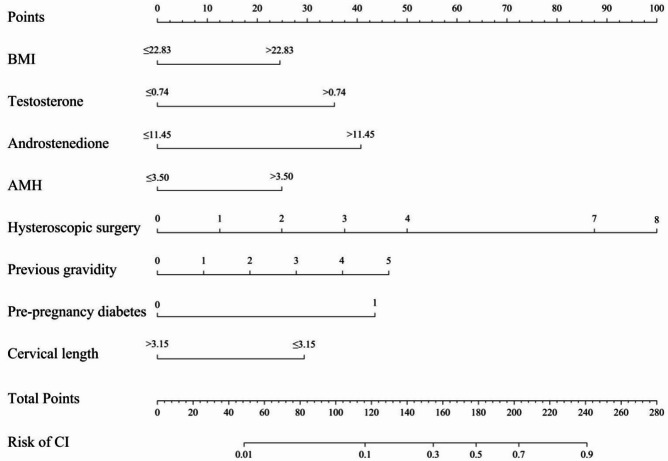
Prediction model for CI, incorporating BMI > 22.83 kg/m2, testosterone level > 0.74mmol/L, androstenedione level > 11.45nmol/L, AMH level > 3.50ng/ml, frequency of hysteroscopic surgery, pre-pregnancy diabetes, cervical length > 3.15 cm and number of previous gravidity as risk factors.

Based on the findings from the univariate, LASSO, and multivariate regression analyses, we developed a predictive model for CI, as shown in Fig. 1. This model incorporates the following risk factors: BMI > 22.83 kg/m², testosterone level > 0.74 mmol/L, androstenedione level > 11.45 nmol/L, AMH level > 3.50 ng/ml, frequency of hysteroscopic surgeries, presence of prepregnancy diabetes and the number of previous pregnancies. Cervical length > 3.15 cm is a protective factor for CI. Each factor is assigned a corresponding score, with cumulative scoring used to estimate the probability of CI occurrence (see Fig. 1).

### Accuracy of the model

**Fig. 2:**
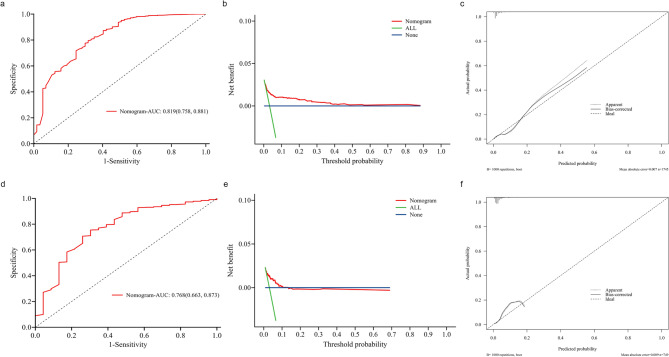
Discrimination and calibration of a model to predict CI in women undergoing IVF-ET treatment. (**a**) ROC curve of a model in training set to predict CI rate in patients undergoing IVF-ET. (**b)** The DCA curve of the training set. (**c)** Calibration curve of training set showing the association between the probability of CI as predicted by the model and the observed CI rate. (**d**) ROC curve of a model in validation set to predict CI rate in patients undergoing IVF-ET. (**e)** The DCA curve of the validation set. (**f**) Calibration curve of validation set showing the association between the probability of CI as predicted by the model and the observed CI rate. Abbreviations: IVF-ET, in vitro fertilization and embryo tranfer; CI, cervical insufficiency; ROC, Receiver Operating Characteristic Curve; DCA: Decision Curve Analysis.

The predictive model demonstrated good performance, with an area under the curve (AUC) of 0.819 in the training cohort. The 95% confidence interval ranged from 0.758 to 0.881, indicating strong discriminatory ability (Fig. 2). The AUC of the validation set was 0.768 (95% confidence interval: 0.662 ~ 0.862), which indicated fair performance (Fig. 2).

The optimal cutoff value of the risk score was determined by maximizing the Youden index to achieve the best balance between sensitivity and specificity. In the training set, this threshold was identified as 84.523, yielding a sensitivity of 68.4%, a specificity of 79.5%, and an AUC of 0.819 (95% CI: 0.758 ~ 0.881). The corresponding ROC curve and decision curve analysis (DCA) are presented in Supplementary Fig. 1.

## Discussion

In the present study, we developed a predictive model for CI based on data from women who underwent IVF-ET treatment at our hospital and subsequently delivered at our facility.

The incidence of CI in this cohort was 3.2%, higher than the rate reported in the general population of women of childbearing age, which is consistent with findings from previous studies^11,16^.

A 2020 study involving 4,710 IVF/ICSI cases developed a CI prediction model and identified factors such as BMI >23.9 kg/m², uterine length ≤ 4.5 cm, gravidity, and testosterone levels >0.7 ng/ml as being associated with an increased risk of CI^17^. Previous studies have demonstrated the utility of hormone levels in predicting clinical pregnancy rates among IVF/ICSI populations. Accordingly, we incorporated hormone level assessments into the design of our study^18^. Compared to earlier research, our study expanded the scope of analysis by incorporating additional variables, such as AMH and androstenedione levels, specific IVF-ET treatment protocols (e.g., embryo cryopreservation), controlled ovarian stimulation strategies for fresh embryo transfer, endometrial preparation methods for frozen embryo transfer, and comorbid conditions like prepregnancy diabetes and hypertension. Furthermore, we considered not only the frequency of uterine cavity operations but also the time interval between the last intrauterine operation and subsequent pregnancy.

By incorporating these comprehensive factors into our predictive model, we aimed to provide a more robust and reliable tool for estimating the risk of CI. This model has the potential to enhance early detection for women at risk of CI.

Previous research has highlighted the association between BMI and adverse pregnancy outcomes, BMI exceeding 30 kg/m² is linked to an elevated risk of premature delivery^19^. Additionally, BMI >23.9 kg/m² as a significant high-risk factor^17^. Our findings are consistent with these observations, as we identified an increased risk of CI at a BMI threshold of >22.83 kg/m².

A potential explanation for this association is that a higher BMI increases abdominal pressure, which, in turn, exerts additional stress on the cervix, potentially leading to the development of CI. Furthermore, the altered endocrine environment observed in women with higher BMIs compared to those with lower BMIs may also contribute to the development of CI.

Hyperandrogenism is recognized as a significant risk factor for CI^20^. This study found that a testosterone level >0.74 nmol/L was significantly associated with an increased risk of CI. This result is largely consistent with the conclusion reported in previous studies that a testosterone level >0.7 ng/ml is associated with an increased risk of CI^17^, further supporting the reasonableness and potential reproducibility of the findings in this study. Additionally, the study also revealed that an androstenedione level >11.5 ng/dl was significantly associated with an increased risk of CI. Given that the research center in this study set >11.5 ng/dl of androstenedione as the clinical reference threshold for hyperandrostenedonism, the conclusion of this study not only has statistical significance but also has certain clinical basis and interpretability. While the exact physiological and pathological mechanisms linking hyperandrogenism to CI remain unclear, androgen metabolism may contribute to reduced cervical collagen tissue^21–23^, which could increase susceptibility to CI.

Current research on the relationship between androgen levels and CI has primarily focused on testosterone. Due to the constraints of detection methods, clinical assessments generally measure total testosterone rather than free testosterone, which is more biologically active. This limitation may reduce the sensitivity of testosterone as a diagnostic marker for hyperandrogenism. Despite this, testosterone exhibits higher biological activity compared to androstenedione^24,25^. Recently, androstenedione has gained recognition in clinical practice for its enhanced sensitivity in diagnosing hyperandrogenism. In this study, both testosterone and androstenedione were identified as independent risk factors for CI through multivariate analysis. Notably, this is the first study to reveal an association between androstenedione levels and CI, highlighting its potential as a valuable marker for assessing CI risk.

AMH is secreted primarily by ovarian granulosa cells^26^ and serves as the most sensitive biomarker for assessing ovarian reserve. A growing body of evidence indicates that serum AMH concentrations are significantly and positively associated with antral follicle count (AFC), while reduced AMH levels are linked to lower clinical pregnancy rates among infertile women^27,28^. AMH has also been identified as a predictor of premature delivery in individuals with PCOS^29–31^. However, the relationship between AMH and CI has not been explored in the current literature. This study proposes that AMH may represent a potential risk factor for CI. The obtained threshold of AMH (>3.50 ng/ml) is similar to the AMH levels of the PCOS preterm population in previous studies^29–31^, indicating that the conclusion of this study has clinical plausibility.

AMH, a member of the TGF-β superfamily, binds to specific receptors and activates the TGF-β signaling pathway.The TGF-β pathway is well known for its roles in fibrosis, cell proliferation, and apoptosis. Previous research has shown that in cervical cancer, AMH can influence cancer cell apoptosis by modulating cell cycle progression^32^. Notably, AMH receptors have been identified in cervical tissues^33^.

Women with impaired fasting glucose or diabetes prior to pregnancy face an increased risk of spontaneous abortion and preterm delivery. Although previous study found no significant correlation between CI and prepregnancy fasting glucose levels^34^, our findings demonstrated a positive association between diabetes and the incidence of CI. Meanwhile, among women with PCOS who experience CI, those with concurrent insulin resistance have poorer pregnancy outcomes^10^. Hyperglycaemia may contribute to degradation of cervical collagen integrity, as diabetes is associated with abnormalities in angiogenesis, cell adhesion, migration, proliferation, differentiation, and extracellular matrix (ECM) deposition^35,36^. Furthermore, studies have shown that diabetes impairs insulin signaling in fibroblasts, resulting in delayed wound healing^37^. These mechanisms warrant further investigation specifically in cervical fibroblasts.

Several risk factors identified in this study-including obesity, elevated androgen levels, high AMH levels, and diabetes-are also well-recognized contributors to PCOS^38^, which itself is considered a significant risk factor for CI. In our analysis, PCOS was positively correlated with CI in univariate regression analysis, but no significant correlation was found in multivariate analyses. This finding is reasonable, as the risk of CI among patients with PCOS likely stems from the presence of these associated risk factors. Excluding PCOS from the predictive model reduces the potential for double counting of risk values, enhancing the model’s reliability.

Our findings suggest that uterine length is not correlated with CI, which contrasts with previous studies that have linked uterine length to premature delivery, abortion^39^, and CI^17^. However, we identified a negative correlation between cervical length more than 3.15 cm prior to pregnancy and CI.

In addition to congenital factors such as collagen disorders, uterine malformations, and cervical length, acquired abnormalities in the cervical anatomy may also contribute to CI. The frequency of hysteroscopy emerged as a high-risk factor for CI. In contrast, the number of curettage procedures did not show a significant relationship with CI outcomes. This lack of association could be attributed to the minimal requirements for cervical dilation during curettage in clinical practice.

Previous research has reported that women undergoing hysteroscopic surgery within six months before pregnancy experience higher rates of miscarriage or premature delivery^40^. However, our study did not identify a significant correlation between CI incidence and intrauterine procedures performed within this timeframe.

Hysteroscopic surgery presents both advantages and disadvantages. While it is associated with an elevated risk of CI, it also improves clinical pregnancy rates. Additionally, hysteroscopic procedures provide an opportunity to evaluate cervical function, particularly in patients with cervical laxity. Given these dual effects, the criteria for performing hysteroscopic surgery should be carefully considered. When operative indications are present, instruments requiring minimal cervical dilation are recommended to mitigate the risk of CI.

Previous studies have reported a higher incidence of CI among women undergoing IVF-ET^11,15^. In this study, we found the incidence of CI to be 3.2% among patients treated with IVF-ET, slightly exceeding the reported incidence in the general population. Several factors may contribute to this finding. First, women undergoing IVF-ET are often affected by a higher prevalence of endocrine disorders, such as PCOS, which may increase the risk of CI. Second, some patients receiving IVF-ET undergo hysteroscopy prior to embryo transfer, which could also elevate the incidence of CI.

Studies have examined pregnancy outcomes associated with fresh and frozen embryo transfers. For instance, Shi Yuhua^41^ reported that compared to fresh embryo transfers, frozen embryo transfers significantly reduced the risk of pregnancy loss in women with PCOS, though CI incidence was not specifically addressed. In contrast, another study^42^ observed a higher incidence of CI in cases involving frozen embryo transfers compared to fresh embryo transfers. In our study, we investigated factors related to infertility and IVF-ET protocols, including methods of embryo cryopreservation and strategies for controlled ovarian stimulation or endometrial preparation. However, no significant association between IVF-ET protocols and the incidence of CI was identified.

We successfully developed a comprehensive predictive model for assessing CI risk in the IVF-ET population. The cutoff value of the total risk score of this model is 84.523, indicating that individuals with a score of ≥ 84.523 are at high risk of CI. It is recommended to enhance the monitoring of cervical length during pregnancy for this group. However, this study has several limitations. First, as a retrospective study, our findings require prospective validation and the lack of an independent external validation cohort may restrict the generalizability of the observed associations. This statistical limitation highlights the critical need for external validation in an expanded multicenter cohort to ensure the robustness of the model. Second, the absence of specific clinical data, such as the duration of hysteroscopy or the type of instruments used, limited our ability to explore the relationship between these factors and CI.

## Conclusion

In conclusion, this article develop a predictive model for CI that includes BMI, testosterone level, androstenedione level, AMH level, frequency of hysteroscopic surgery, cervical length as risk factors. For the first time, AMH and androstenedione have been incorporated as predictors in a CI prediction model, Our model suggests potential utility for identifying CI risk in IVF-ET patients, incorporating AMH and androstenedione as exploratory predictors. It is also found that different IVF-ET protocols were not associated with CI. By employing this predictive tool, we can more accurately identify high-risk groups for CI prior to pregnancy. This advancement enhances the monitoring of cervical length during gestation, facilitating timely interventions that may mitigate the risks of miscarriage or preterm birth associated with CI.

## Supplementary Information

Below is the link to the electronic supplementary material.

Supplementary Material 1

## Data Availability

The datasets used and/or analyzed during the current study are available from the corresponding author on reasonable request due to data related to patient privacy.

## References

[1] Guo, Y. Wang Yong-qing. Confusion in diagnosis and treatment of cervical insufficiency[J]. *Int. Reprod. Health/Fam Plan.* **37** (05), 417–421 (2018).

[2] DaCosta, V. et al. Laparoscopic cervicoisthmic cerclage for the treatment of cervical incompetence: case reports. *West. Indian Med. J.* **60** (5), 590–593 (2011). 22519240

[3] Brown, R., Gagnon, R. & Delisle, M. F. 373-Cervical insufficiency and cervical cerclage. *J. Obstet. Gynaecol. Can.* **41** (2), 233–247 (2019). 30638557 10.1016/j.jogc.2018.08.009

[4] Goldenberg, R. L., Culhane, J. F., Iams, J. D. & Romero, R. Epidemiology and causes of preterm birth. *Lancet* **371** (9606), 75–84 (2008). 18177778 10.1016/S0140-6736(08)60074-4 PMC7134569

[5] Roman, A., Suhag, A. & Berghella, V. Overview of cervical insufficiency: diagnosis, etiologies, and risk factors. *Clin. Obstet. Gynecol.* **59**, 23–40 (2016). 10.1097/GRF.0000000000000184 27015229

[6] Han, Y., Li, M., Ma, H. & Yang, H. Cervical insufficiency: a noteworthy disease with controversies. *J. Perinat. Med.* **48** (7), 648–655 (2020). 32692707 10.1515/jpm-2020-0255

[7] Gascón, A. et al. Cervical cerclage vs cervical Pessary in women with cervical insufficiency: A multicentric, open-label, randomised, controlled pilot trial [the CEPEIC trial]. *Eur. J. Obstet. Gynecol. Reprod. Biol. X*. **24**, 100347 (2024). 39497908 10.1016/j.eurox.2024.100347 PMC11532433

[8] Zhu, L. Q. et al. Effects of emergency cervical cerclage on pregnancy outcome: a retrospective study of 158 cases. *Med. Sci. Monit.* **21**, 1395–1401 (2015). 25975832 10.12659/MSM.893244 PMC4444177

[9] Chen, Q., Chen, G. & Li, N. Clinical effect of emergency cervical cerclage and elective cervical cerclage on pregnancy outcome in the cervical-incompetent pregnant women. *Arch. Gynecol. Obstet.* **297** (2), 401–407 (2018). 29222640 10.1007/s00404-017-4602-7

[10] Wang, Y., Gu, X., Tao, L. & Zhao, Y. Co-morbidity of cervical incompetence with polycystic ovarian syndrome (PCOS) negatively impacts prognosis: A retrospective analysis of 178 patients. *BMC Pregnancy Childbirth*. **16** (1), 308 (2016). 27733131 10.1186/s12884-016-1094-6 PMC5062886

[11] Feigenbaum, S. L. et al. Prevalence of cervical insufficiency in polycystic ovarian syndrome. *Hum. Reprod.* **27** (9), 2837–2842 (2012). 22698930 10.1093/humrep/des193 PMC3415288

[12] Meng, L., Öberg, S., Sandström, A., Wang, C. & Reilly, M. Identification of risk factors for incident cervical insufficiency in nulliparous and Parous women: a population-based case-control study. *BMC Med.* **20** (1), 348 (2022). 36221132 10.1186/s12916-022-02542-7 PMC9555073

[13] Anum, E. A., Hill, L. D., Pandya, A. & Strauss, J. F. 3rd Connective tissue and related disorders and preterm birth: clues to genes contributing to prematurity. *Placenta* **30** (3), 207–215 (2009). 19152976 10.1016/j.placenta.2008.12.007 PMC2673455

[14] Spiegel, E., Nicholls-Dempsey, L., Czuzoj-Shulman, N. & Abenhaim, H. A. Pregnancy outcomes in women with Ehlers-Danlos syndrome. *J. Matern Fetal Neonatal Med.* **35** (9), 1683–1689 (2022). 32654548 10.1080/14767058.2020.1767574

[15] Richard, D. et al. Calculating the sample size required for developing a clinical prediction model. *BMJ* **368**, m441 (2020). 32188600 10.1136/bmj.m441

[16] Meng, L., Öberg, S., Sandström, A. & Reilly, M. Association between infertility and cervical insufficiency in nulliparous women- the contribution of fertility treatment. *Am. J. Obstet. Gynecol.* **28**, S0002-9378(24)01107-4. 10.1016/j.ajog.2024.10.035 39477049

[17] Wu, Y. et al. Development and validation of a model for individualized prediction of cervical insufficiency risks in patients undergoing IVF/ICSI treatment. *Reprod. Biol. Endocrinol.* **19** (1), 6 (2021). 33413472 10.1186/s12958-020-00693-x PMC7789534

[18] Sun, X. et al. Independent value of PMOI on hCG day in predicting pregnancy outcomes in IVF/ICSI cycles. *Front. Endocrinol. (Lausanne)*. **14**, 1086998 (2023). 36909315 10.3389/fendo.2023.1086998 PMC9997210

[19] Liu, B. et al. Association between maternal pre-pregnancy obesity and preterm birth according to maternal age and race or ethnicity: a population-based study. *Lancet Diabetes Endocrinol.* **7** (9), 707–714 (2019). 31395506 10.1016/S2213-8587(19)30193-7 PMC6759835

[20] Shetelig Løvvik, T., Stridsklev, S., Carlsen, S. M., Salvesen, Ø. & Vanky, E. Cervical length and androgens in pregnant women with polycystic ovary syndrome: has Metformin any effect? *J. Clin. Endocrinol. Metab.* **101** (6), 2325–2331 (2016). 26835542 10.1210/jc.2015-3498

[21] Makieva, S., Saunders, P. T. & Norman, J. E. Androgens in pregnancy: roles in parturition[J]. *Hum. Reprod. Update*. **20** (4), 542–559 (2014). 24643344 10.1093/humupd/dmu008 PMC4063701

[22] Sundtoft, I., Langhoff-Roos, J., Sandager, P., Sommer, S. & Uldbjerg, N. Cervical collagen is reduced in non-pregnant women with a history of cervical insufficiency and a short cervix. *Acta Obstet. Gynecol. Scand.* **96** (8), 984–990 (2017). 28374904 10.1111/aogs.13143

[23] Timmons, B., Akins, M. & Mahendroo, M. Cervical remodeling during pregnancy and parturition. *Trends Endocrinol. Metab.* **21** (6), 353–361 (2010). 20172738 10.1016/j.tem.2010.01.011 PMC2880223

[24] Biffignandi, P., Massucchetti, C. & Molinatti, G. M. Female hirsutism: pathophysiological considerations and therapeutic implications. *Endocr. Rev.* **5** (4), 498–513 (1984). 6094172 10.1210/edrv-5-4-498

[25] Wu Jing, L. & Ruojia, C. Significance of different androgen indexes in the diagnosis of hyperandrogenemia in polycystic ovary syndrome. *Zhejiang Med. J.* **43** (5), 537–539 (2021).

[26] Pankhurst, M. W. A putative role for anti-Mullerian hormone (AMH) in optimising ovarian reserve expenditure. *J. Endocrinol.* **233** (1), R1–R13 (2017). 28130407 10.1530/JOE-16-0522

[27] Sun, X. et al. Comparison of the predictive capability of antral follicle count vs. the anti-Müllerian hormone for ovarian response in infertile women. *Front. Endocrinol. (Lausanne)*. **13**, 862733 (2022). 36387919 10.3389/fendo.2022.862733 PMC9659916

[28] Sun, X. Y. et al. Relationship between Anti-Müllerian hormone and in vitro Fertilization-Embryo transfer in clinical pregnancy. *Front. Endocrinol. (Lausanne)*. **11**, 595448 (2020). 33343511 10.3389/fendo.2020.595448 PMC7746804

[29] Hu, K. L., Liu, F. T., Xu, H., Li, R. & Qiao, J. High antimüllerian hormone levels are associated with preterm delivery in patients with polycystic ovary syndrome. *Fertil. Steril.* **113** (2), 444–452e1 (2020). 31973904 10.1016/j.fertnstert.2019.09.039

[30] Hsu, J. Y. et al. Mullerian-Inhibiting Substance/Anti-Mullerian hormone as a predictor of preterm birth in polycystic ovary syndrome. *J. Clin. Endocrinol. Metab.* **103** (11), 4187–4196 (2018). 30239805 10.1210/jc.2018-01320

[31] Kaing, A. et al. Highly elevated level of antimüllerian hormone associated with preterm delivery in polycystic ovary syndrome patients who underwent ovulation induction. *Fertil. Steril.* **115** (2), 438–446 (2021). 32883514 10.1016/j.fertnstert.2020.06.015

[32] Kang, J. *Molecular Mechaisms of Mouse Mullerian Duct development[D]* (Beijing, Beijing Xiehe Medical College Hospital, Chinese Academy of Medical Sciences,, 2020).

[33] Song, J. Y. et al. Expression of Müllerian inhibiting substance type II receptor and antiproliferative effects of MIS on human cervical cancer. *Int. J. Oncol.* **40** (6), 2013–2021 (2012). 22344630 10.3892/ijo.2012.1370 PMC5609185

[34] Wei, Y. et al. Preconception diabetes mellitus and adverse pregnancy outcomes in over 6.4 million women: A population-based cohort study in China. *PLoS Med.* **16** (10), e1002926 (2019). 31574092 10.1371/journal.pmed.1002926 PMC6771981

[35] Michalik, L. & Wahli, W. Involvement of PPAR nuclear receptors in tissue injury and wound repair. *J. Clin. Invest.* **116** (3), 598–606 (2006). 16511592 10.1172/JCI27958 PMC1386118

[36] Werner, S. & Grose, R. Regulation of wound healing by growth factors and cytokines. *Physiol. Rev.* **83** (3), 835–870 (2003). 12843410 10.1152/physrev.2003.83.3.835

[37] Khamaisi, M. et al. PKCδ Inhibition normalizes the wound-healing capacity of diabetic human fibroblasts. *J. Clin. Invest.* **126** (3), 837–853 (2016). 26808499 10.1172/JCI82788 PMC4767341

[38] Li, R. et al. Prevalence of polycystic ovary syndrome in women in china: A large Community-Based study. *Hum. Reprod.* **28** (9), 2562–2569 (2013). 23814096 10.1093/humrep/det262

[39] Hawkins, L. K., Correia, K. F., Srouji, S. S., Hornstein, M. D. & Missmer, S. A. Uterine length and fertility outcomes: a cohort study in the IVF population. *Hum. Reprod.* **28** (11), 3000–3006 (2013). 24014604 10.1093/humrep/det344

[40] Gökçe, A. et al. The association between operative hysteroscopy prior to assisted reproductive technology and cervical insufficiency in second trimester. *Arch. Gynecol. Obstet.* **303** (5), 1347–1352 (2021). 33219481 10.1007/s00404-020-05863-1

[41] Shi, Y. et al. Transfer of fresh versus frozen embryos in ovulatory women. *N Engl. J. Med.* **378** (2), 126–136 (2018). 29320646 10.1056/NEJMoa1705334

[42] Liu, Y., Li, R. & Wang, Y. Clinical outcomes and placental pathological characteristics after fresh embryo transfer and frozen-thawed embryo transfer with different endometrial Preparation protocols. *Placenta* **131**, 65–70 (2023). 36493625 10.1016/j.placenta.2022.11.011

