# Supplementary material for: Development and validation of a predictive model for cervical insufficiency incorporating AMH and androstenedione
Source: Sci Rep. 2025 Dec 13;16:2022. doi: 10.1038/s41598-025-31678-8 (PMC12808648; doi:10.1038/s41598-025-31678-8)
Supplement: Supplementary file 1 — Supplementary Material 1 [file 41598_2025_31678_MOESM1_ESM.docx]

**Supplement Table 1**: Characteristics in the training and the validation cohort

|  | **Total** | **Training set** | **Validation set** | **P** |
| --- | --- | --- | --- | --- |
|  | **N=2494** | **N=1745** | **N=749** |  |
| Age, y | 34.00 [31.00;36.00] | 34.00 [31.00;36.00] | 34.00 [31.00;36.00] | 0.941 |
| BMI, kg/m^2^, n (%) |  |  |  | 0.510 |
| ≤22.83 | 1609 (64.5) | 1133 (64.9) | 476 (63.6) |  |
| >22.83 | 885 (35.5) | 612 (35.1) | 273 (36.4) |  |
| LH/FSH, n (%) |  |  |  | 0.172 |
| ​≤1.03 | 237 (9.5) | 175 (10.0) | 62 (8.3) |  |
| ​>1.03 | 2257 (90.5) | 1570 (90.0) | 687 (91.7) |  |
| E2, pmol/L, n (%) |  |  |  | 0.487 |
| ​≤175.50 | 1469 (58.9) | 1020 (58.5) | 449 (59.9) |  |
| ​>175.50 | 1025 (41.1) | 725 (41.5) | 300 (40.1) |  |
| P, nmol/L, n (%) |  |  |  | 0.033 |
| ​≤1.19 | 1224 (49.1) | 832 (47.7) | 392 (52.3) |  |
| ​>1.19 | 1270 (50.9) | 913 (52.3) | 357 (47.7) |  |
| T, nmol/L, n (%) |  |  |  | 0.952 |
| ​≤0.74 | 1876 (75.2) | 1312 (75.2) | 564 (75.3) |  |
| ​>0.74 | 618 (24.8) | 433 (24.8) | 185 (24.7) |  |
| A, nmol/L, n (%) |  |  |  | 0.316 |
| ​≤11.45 | 2314 (92.8) | 1625 (93.1) | 689 (92) |  |
| ​>11.45 | 180 (7.2) | 120 (6.9) | 60 (8) |  |
| AMH, ng/ml, n (%) |  |  |  | 0.566 |
| ​≤3.50 | 1768 (70.9) | 1243 (71.2) | 525 (70.1) |  |
| ​>3.50 | 726 (29.1) | 502 (28.8) | 224 (29.9) |  |
| PCOS, n (%) |  |  |  | 0.741 |
| NO | 2232 (89.5) | 1564 (89.6) | 668 (89.2) |  |
| YES | 262 (10.5) | 181 (10.4) | 81 (10.8) |  |
| Embryo cryopreservation, n (%) | | | | 0.382 |
| Frozen embryo | 1152 (46.2) | 816 (46.8) | 336 (44.9) |  |
| Fresh embryo | 1342 (53.8) | 929 (53.2) | 413 (55.1) |  |
| Protocol of controlled ovarian stimulation/Endometrial preparation plan, n (%) | | | | 0.370 |
| CC | 3 (0.1) | 2 (0.1) | 1 (0.1) |  |
| Ultralong GnRH agonist | 187 (7.5) | 118 (6.8) | 69 (9.2) |  |
| Long GnRH agonist | 295 (11.8) | 203 (11.6) | 92 (12.3) |  |
| Short GnRH agonist | 20 (0.8) | 14 (0.8) | 6 (0.8) |  |
| GnRH agonist | 837 (33.6) | 592 (33.9) | 245 (32.7) |  |
| Natural cycle | 598 (24) | 431 (24.7) | 167 (22.3) |  |
| Artificial cycle | 554 (22.2) | 385 (22.1) | 169 (22.6) |  |
| Frequency of hysteroscopic surgery | 0.00 [0.00, 1.00] | 0.00 [0.00, 1.00] | 0.00 [0.00, 1.00] | 0.297 |
| Frequency of curettage operation | 0.00 [0.00, 1.00] | 0.00 [0.00, 1.00] | 0.00 [0.00, 1.00] | 0.098 |
| Last intrauterine operation within 6 months from the last menstruation | | | | 0.636 |
| No | 983 (39.4) | 678 (38.9) | 305 (40.7) |  |
| Yes | 422 (16.9) | 301 (17.2) | 121 (16.2) |  |
| No operation | 1089 (43.7) | 766 (43.9) | 323 (43.1) |  |
| G | 0.00 [0.00, 1.00] | 0.00 [0.00, 1.00] | 0.00 [0.00, 1.00] | 0.037 |
| P |  |  |  | 0.648 |
| 0, n (%) | 2336 (93.7) | 1637 (93.8) | 699 (93.3) |  |
| ≧1, n (%) | 158 (6.3) | 108 (6.2) | 50 (6.7) |  |
| Twin pregnancy, n (%) |  |  |  | 0.003 |
| No | 1969 (78.9) | 1405 (80.5) | 564 (75.3) |  |
| Yes | 525 (21.1) | 340 (19.5) | 185 (24.7) |  |
| Prepregnancy diabetes, n (%) |  |  |  | 0.158 |
| No | 2442 (97.9) | 1704 (97.7) | 738 (98.5) |  |
| Yes | 52 (2.1) | 41 (2.3) | 11 (1.5) |  |
| Prepregnancy hypertension, n (%) | | |  | 0.620 |
| No | 2428 (97.4) | 1697 (97.2) | 731 (97.6) |  |
| Yes | 66 (2.6) | 48 (2.8) | 18 (2.4) |  |
| Uterine length, cm, n (%) |  |  |  | 0.960 |
| ​≤4.95 | 1457 (58.4) | 1020 (58.5) | 437 (58.3) |  |
| ​>4.95 | 1037 (41.6) | 725 (41.5) | 312 (41.7) |  |
| Cervical length, cm, n (%) |  |  |  | 0.530 |
| ​≤3.15 | 1588 (63.7) | 1118 (64.1) | 470 (62.8) |  |
| ​>3.15 | 906 (36.3) | 627 (35.9) | 279 (37.2) |  |

**Supplement Table 2**: LASSO regression screening predictors

| Predictors |  |
| --- | --- |
| BMI>22.83, kg/m^2^ | 0.270064629 |
| Testosterone>0.74, nmol/L | 0.704966658 |
| Androstenedione>11.45, nmol/L | 1.238894845 |
| AMH>3.50, ng/ml | 0.18913382 |
| PCOS | 0.378188224 |
| Frequency of hysteroscopic surgery | 0.187945633 |
| Previous gravidity | 0.060439953 |
| Pre-pregnancy diabetes | 0.759875024 |
| Prepregnancy hypertension | 0.109539813 |
| Cervical length>3.15,cm | -0.141304704 |


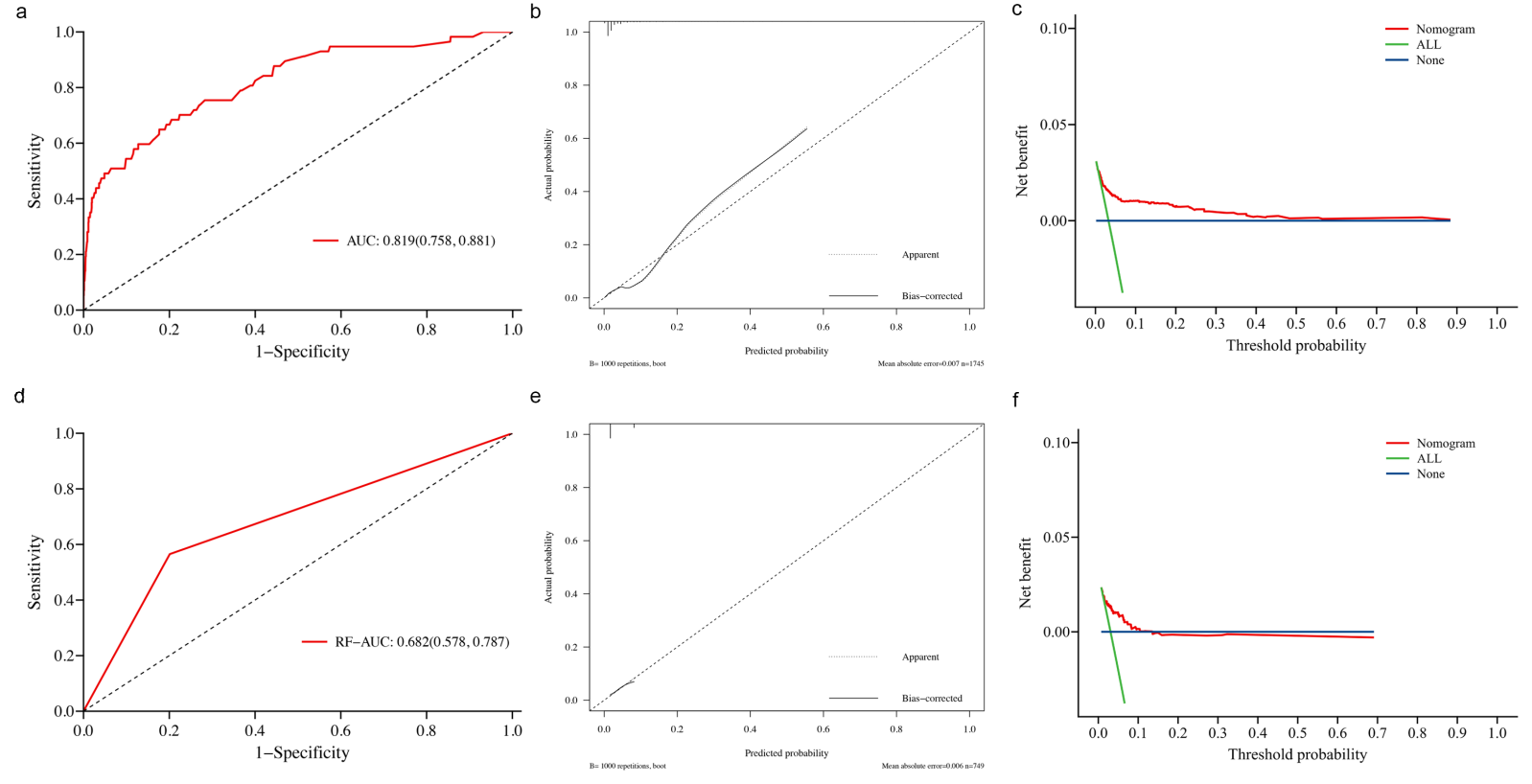


***Supplement Figure 1*** The optimal cutoff value of the risk score was determined by maximizing the Youden index to achieve the best balance between sensitivity and specificity. In the training set, this threshold was identified as 84.523, a: ROC curve of cutoff value in training set to predict cervical insufficiency rate in patients undergoing IVF-ET. b: The calibration curve of the training set. c: The DCA curve of the training set. d:ROC curve of cutoff value in validation set . e: The calibration curve of the validation set. f: The DCA curve of the validation set
